# Temporal and spatial impact of lockdown during COVID-19 on air quality index in Haryana, India

**DOI:** 10.1038/s41598-022-20885-2

**Published:** 2022-11-21

**Authors:** Manjeet, Anurag Airon, Rahul Kumar, Ruksar Saifi

**Affiliations:** 1 Department of Agricultural Meteorology, Chaudhary Charan Singh Haryana Agricultural University, Hisar, Haryana 125004 India; 2 Department of Zoology and Aquaculture, Chaudhary Charan Singh Haryana Agricultural University, Hisar, Haryana 125004 India

**Keywords:** Environmental sciences, Environmental social sciences

## Abstract

This paper presents the evaluation of air quality in different districts of Haryana. Geo-spatial techniques were used to estimate gaseous and particulate pollutant's spatial and temporal variation during complete nationwide lockdown period and same month of previous year 2019 (March to May). Data of six fixed pollutants were collected from Central Pollution Control Board (CPCB). In this context, the data of air pollutants (PM_10_, PM_2.5_, O_3_, NOx, SO_2_, and CO) were analyzed for 2019 and 2020. The Spatio-temporal distribution of the Air Quality Index (AQI) clearly depicts difference in lockdown and unlock period. The result was showed that the air quality was very poor to satisfactory in 2019 and an improvement was observed from satisfactory to good in 2020 due to COVID-19 lockdown. On the basis of result, it will be concluded that automobile and industry are the major contributor in increase the pollutant concentration.

## Introduction

Air pollution has become a critical threat to the environment as well as caused serious threats to health. According to World Health Organization (WHO), about 80% of people in urban areas are exposed to air pollution and 98% of cities in low-middle income countries and 56% in high-income countries do not meet the WHO guidelines^1^. Due to exposure to ambient air pollution death rate of more than 4.2 million people is estimated^1^. Anthropogenic activities are a major source of air pollution such as transport, industrialization, biomass burning^2^. It is reported that anthropogenic activities contributes approximately 80% increase in the pollution^3^. Thus minimize the human activities would have resulted in reduced the level of air pollutants as observed at the global and regional level during the COVID-19 driven lockdown in 2020^4–11^. The government of India issued an advisory for travellers from China in early January and also started screening the travellers from China. On the basis of increased cases of COVID-19, the Indian Prime Minister announced the Janata curfew on 22 March 2020 from 7 am until 9 pm^12^. After 2 days government of India announced a complete nationwide lockdown, from 24 March to 14 April 2020, all the domestic and international flights, trains, and vehicular transport except for non-essential purposes were stopped and banned^13^. During the complete lockdown in India, roads were deserted without any vehicles except emergency vehicles. The month of April every year is the peak time of winter crop harvesting (wheat) and planting of vegetables in India, so the Government relaxed the movement of farmers from lockdown in the second phase from 15 April to 3 May 2020. The Government of India has further extended lockdown in some parts in a relaxed manner; now they have opened vehicular transport, domestic flights, and few trains; as a result, the air quality is getting poor. Also, farmers at many places have started burning crop residue, and long-term transport of dust during the pre-monsoon season is also being observed, which affects the air quality of Delhi and major cities located in the Indo-Gangetic Plains (IGP). Recently, Sharma et al. considered the Central Pollution Control Board dataset and studied the impact of lockdown on air quality for the period 15 March to 14 April 2020^6^. Based on the analysis of satellite derived, air pollutants for April 2019 and 2020 over Haryana, India showed highest decrease in PM_10_ followed by PM_2.5_, AOD, SO_2_, NO_2_, CO, and CH_4_ and improved air quality (AQI)^14^. The real-time observation of various air pollutants asserts that the different gas emission rates drastically fall during the month of April and May 2020 caused by COVID-19 lockdown. Fossil fuel burning has been recorded in low consumption and daily global CO_2_ emission in April decreased by − 17% compared to the average of 2019^15^. The other countrywide research shows the major air pollutants have drastically fallen during the COVID-19 lockdown improving the air quality^6,16,17^. Fang et al. observed a reduction of 18–45%,17–53%, 47–64%, 9–34%, and 16–52%, respectively for particulate matters 2.5 (PM_2.5_), particulate matters 10 (PM_10_), NO_2_, Sulfur Dioxide (SO_2_) and Carbon Monoxide (CO), over urban agglomerations in China, during lockdown period relative to pre-lockdown period^18^. Mendez- Espinosa et al. reported a reduction of 60%, 44%, and 40% respectively in the concentration of NO_2_, PM_10_, and PM2_.5_ over South America during the strict lockdown amid COVID-19^19^. The aim of this paper is to study the impact of complete lockdown in Haryana on air quality during COVID-19 by comparing air quality parameters from March to April 2019 and 2020.

## Material and methods

### Study area

**Figure 1:**
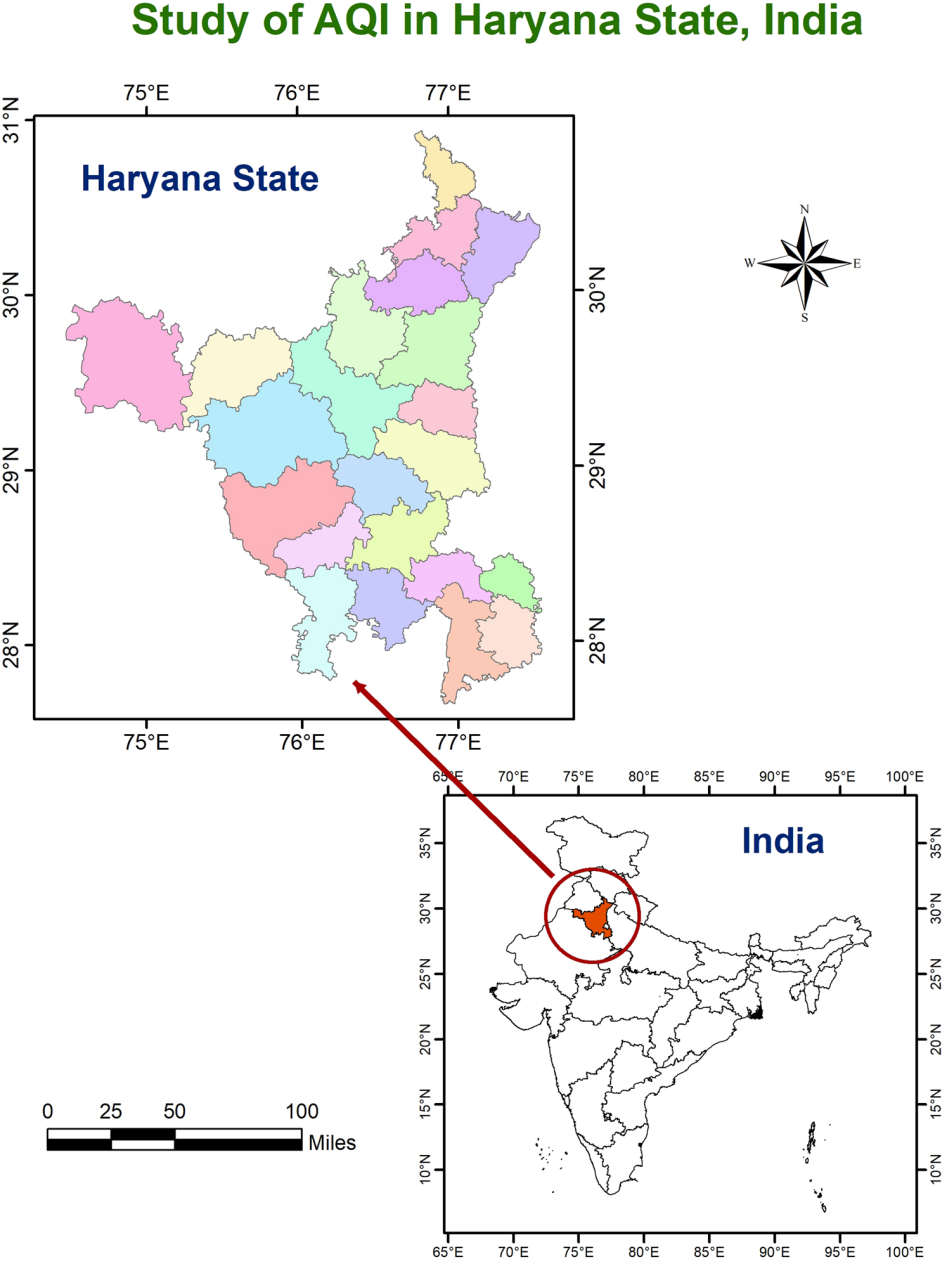
Represent the study location of air quality index measurement.

The present study was conducted in Haryana state located between 27°39′ to 30°35′ N latitude and between 74°28′ and 77°36′ E longitude as shown in Fig. 1. Haryana is a landlocked state in northern India. The study area includes 22 districts of the state with a total area of 44,212 km^2^ i.e. 1.3% of the total area of the country. The Eastern zone includes the district of Panchkula, Ambala, Yamunanagar, Kurukshetra, Karnal, Kaithal, Panipat, Sonipat, Faridabad, and parts of districts of Jind, Rohtak, Jhajjar, and Gurgaon. It covers about 49 percent area of the State which comprises the Gurgaon tract, Rohtak tract, Central plain, and Hill tract. The Western Zone includes the districts of Sirsa, Hisar, Bhiwani, Fatehabad, Mahendragarh, Rewari, and some parts of Jind, Rohtak, Jhajjar, and Gurgaon. There have been rapid developments in the industry, transportation, and the economy. However, the air pollution that comes from development has become a serious matter. The air quality of the state varies from good to severe in a different month of year and seasons. The present investigation and analysis of the temporal and spatial characteristics of air pollutants are therefore important for improving the air quality in Haryana.

### Data sources

The monitoring station data used in this study were obtained from site CPCB (Central Pollution Control Board) and included six air, pollutants, namely, coarse particulate matter (PM_10_), fine particulate matter (PM_2.5_), ozone (O_3_), nitrous oxide (NO_X_), sulfur dioxide (SO_2_) and carbon monoxide (CO)^20^. Data are retrieved every 1 h, which are calculated as the average of the measurements taken every 24 h. One or two district data were not available i.e. Charkhi Daderi (2019), Mewat (2019 &2020), Kaithal.

The AQI is a dimensionless index that quantitatively describes air quality. According to the Central Control Pollution Board, Air Quality Standards implemented by the Ministry of Environment Forest and Climate Change and AQI was calculated by the AQI calculator given by the CPCB site by using the various factor (PM_10_, PM_2.5_, O_3_, NO_x_, SO_2_, and CO).

When AQI greater than 50 represents the major pollutant concentration has increased. There are six AQI categories and their range are 0–50, 51–100, 101–200, 201–300, 301–400, and 401–500. The corresponding pollution levels are good, satisfactory, moderate, poor, very poor, and severe respectively. The higher AQI, the air quality becomes worse.

The mathematical equation for calculating sub-indices of AQI is as follows:

$${\text{Ip }} = \frac{{\left( {{\text{IHI }}{-}{\text{ ILO}}} \right)}}{{\left( {{\text{BPHI }}{-}{\text{ BPLO}}} \right)}} \, \times \, \left( {{\text{CP }} - {\text{ BPLO}}} \right) \, + {\text{ ILO}}$$

where IP is AQI for pollutant “P” (Rounded to the nearest integer), CP the actual ambient concentration of pollutant “P”, BPHI the upper-end breakpoint concentration that is greater than or equal to CP, BPLO the lower end breakpoint concentration that is less than or equal to CP, ILO the sub-index or AQI value corresponding to BPLO, IHI the sub-index or AQI value corresponding to BPHI^21^.


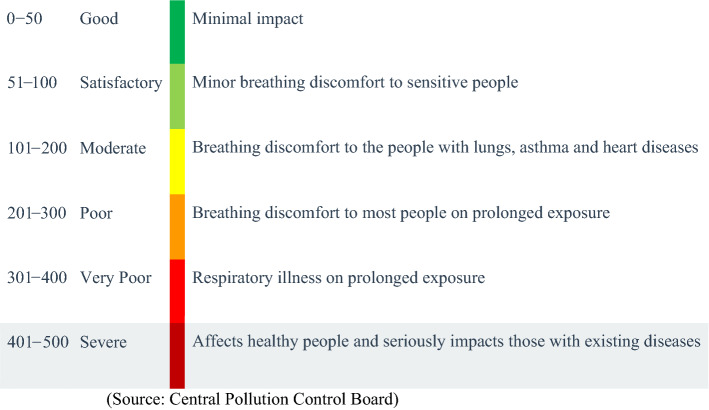


### Data processing

The data were analyzed in MS-Excel by putting daily data of different pollutants. The average of daily data was converted into monthly individual pollutants to calculate the air quality index. Statistical analysis and Arc GIS were used to explore the temporal and spatial quantity of air pollution. For spatial analysis, spatial characterization is conducted through the use of the spatial analysis method with the support of Arc GIS software (Version 10.4 available at Geoinformatic lab, Department of Agricultural Meteorology, Chaudhary Charan Singh, Haryana Agricultural University, Hisar, Haryana (published by the Environmental Systems Research Institute (ESRI), Redlands, California, USA)^22^.

## Result

### Statues of AQI in 2019 during March to May

**Figure 2:**
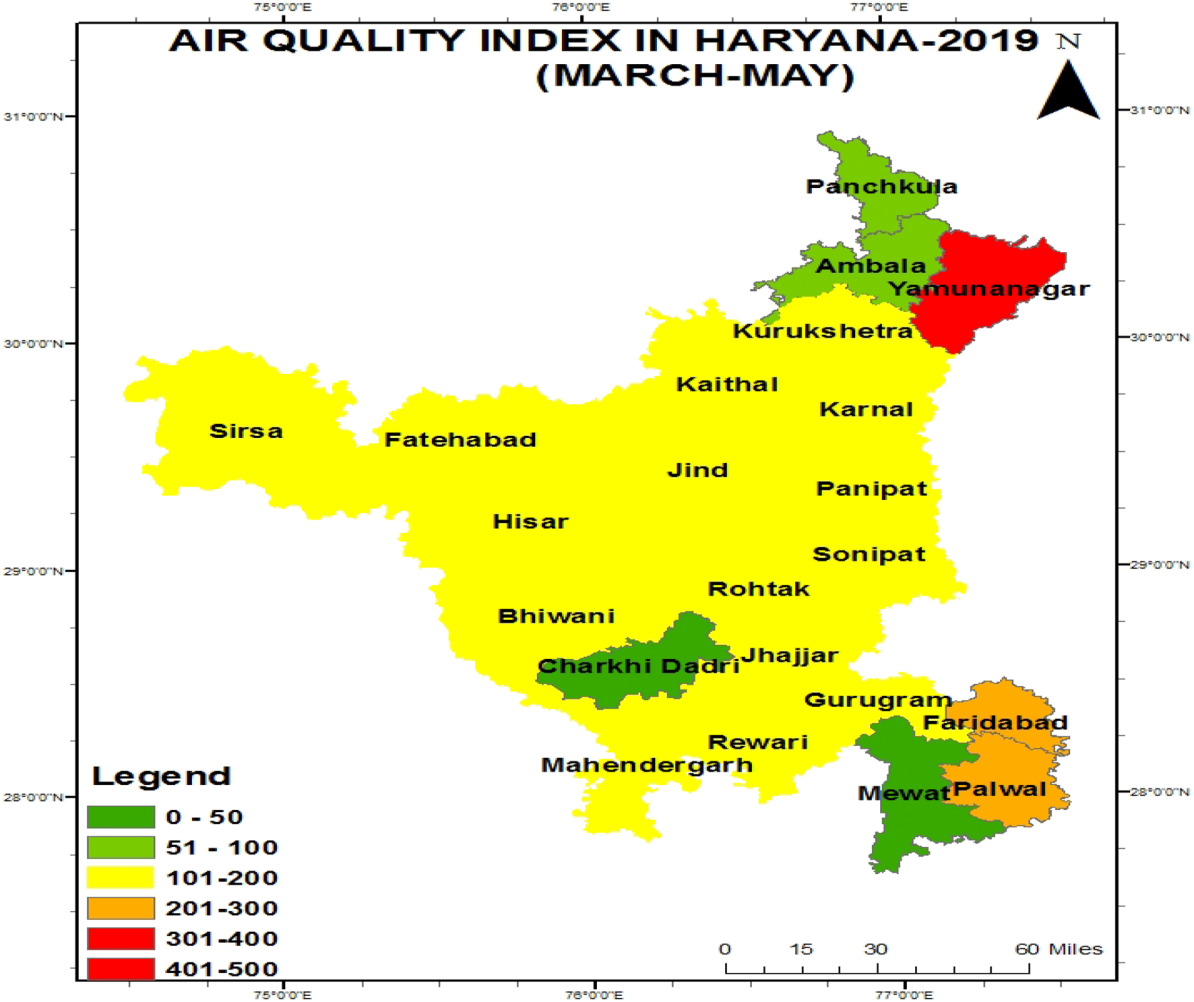
Figure showing the map of Haryana with their respective air quality index during 2019.

Figure 2 shows that from March to May, the AQI was found very poor in Yamuna Nagar, Poor in Faridabad and Palwal, Satisfactory in Panchkula and Ambala and the remaining districts showed moderate-quality except for Mewat and Charkhi Dadari due to data was not available.

### Status of AQI in 2020 during lockdown

**Figure 3:**
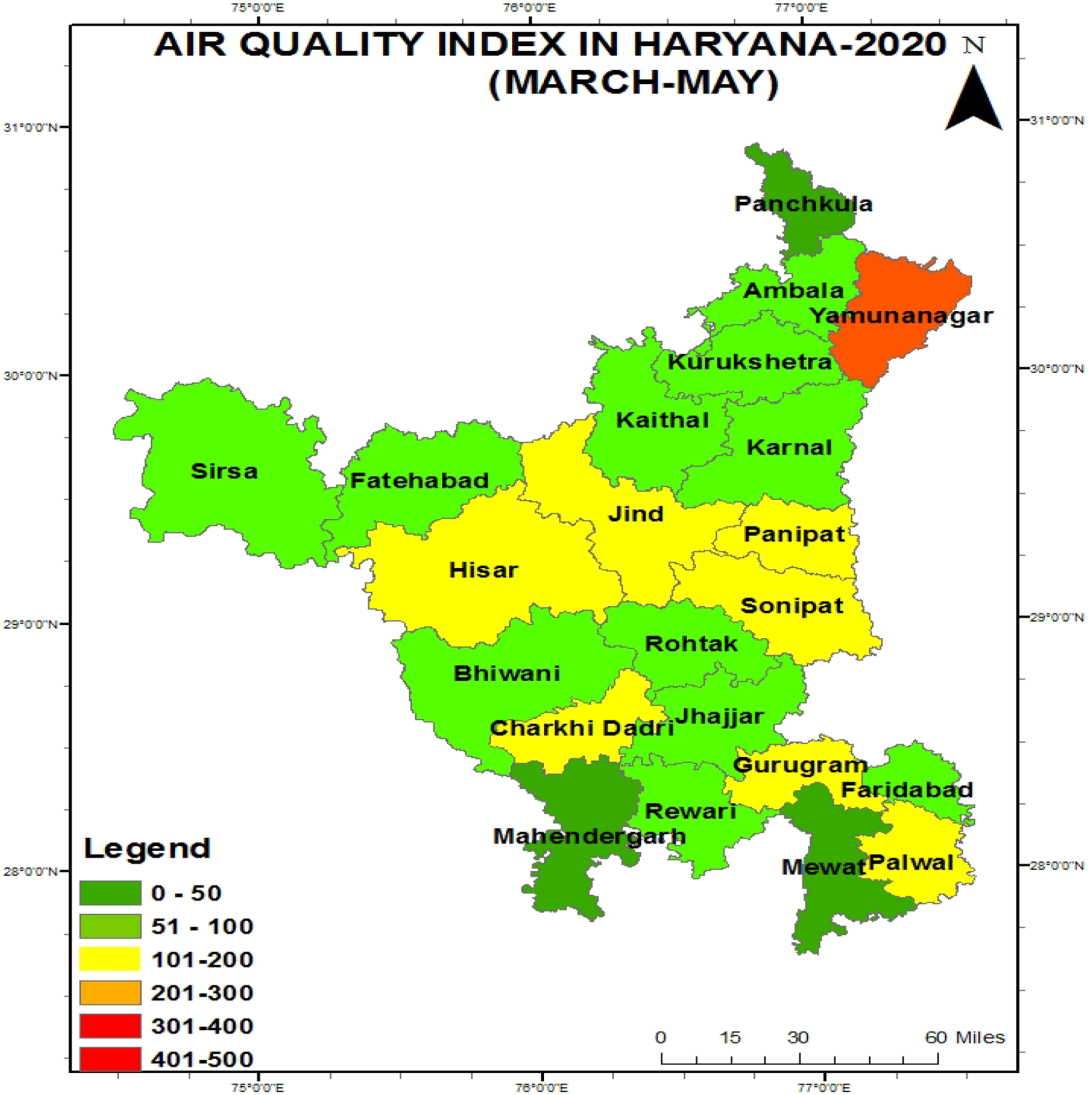
Figure showing the map of Haryana with their respective air quality index during 2020.

Figure 3 shows that from March to May, the AQI was found very poor in Yamuna Nagar, Moderate in Hisar, Jind, Panipat, Sonipat, Charkhi Daderi, Gurugram, Palwal, good in Mahendergarh and the remaining districts showed satisfactory quality except for Mewat due to data was not available during complete lockdown period.

### Impact of pre-lockdown and lockdown on meteorological variables

Temperature is most important meteorological variable for the assessment environmental pollution, air quality index etc. Analyzing the maximum temperature during pre-lockdown and lockdown period. It was observed that temperature showed significant decrease in during lock period as compared to pre-lock period due less emission of green house gas from transportation and industry as data attached in supplementary file (Fig. S1).

### Deviation of air quality index in lockdown period

Table 1 represents the percentage deviation from 21 and above showed shift in air quality index category. In 2020 during lockdown, the air quality index was improved one step from moderate to satisfactory, poor to moderate category. Bhankhar et al. showed that Gurugram is fast growing industry hub and other districts under developing city^23^.

**Table 1:** Depict the change in air quality index.

Percentage deviation category (%)	Districts	AQI (shift)
0–10	Panipat, Charkhi Daderi, Mewat, Yamuna Nagar	No change
11–20	Ambala, Sonipat	No change
21–30	Kurushetra, Kaithal, Sirsa, BhiwaniJajjar,Jind	Moderate to satisfactory (except Jind)
31–40	Panchkula, Hissar,	No change
41–50	Fatehabad, Gurugarm, Rewari	Moderate to satifactroy (except Gurugram)
51–60	Karnal, Rohtak, Palwal	Moderate to satifactory Palwal (poor to moderate)
61–70	Faridabad	Poor to satisfactory
71–80	Mahedergarh	Moderate to good
81–90	Nil	Nil
91–100	Nil	Nil

**Figure 4:**
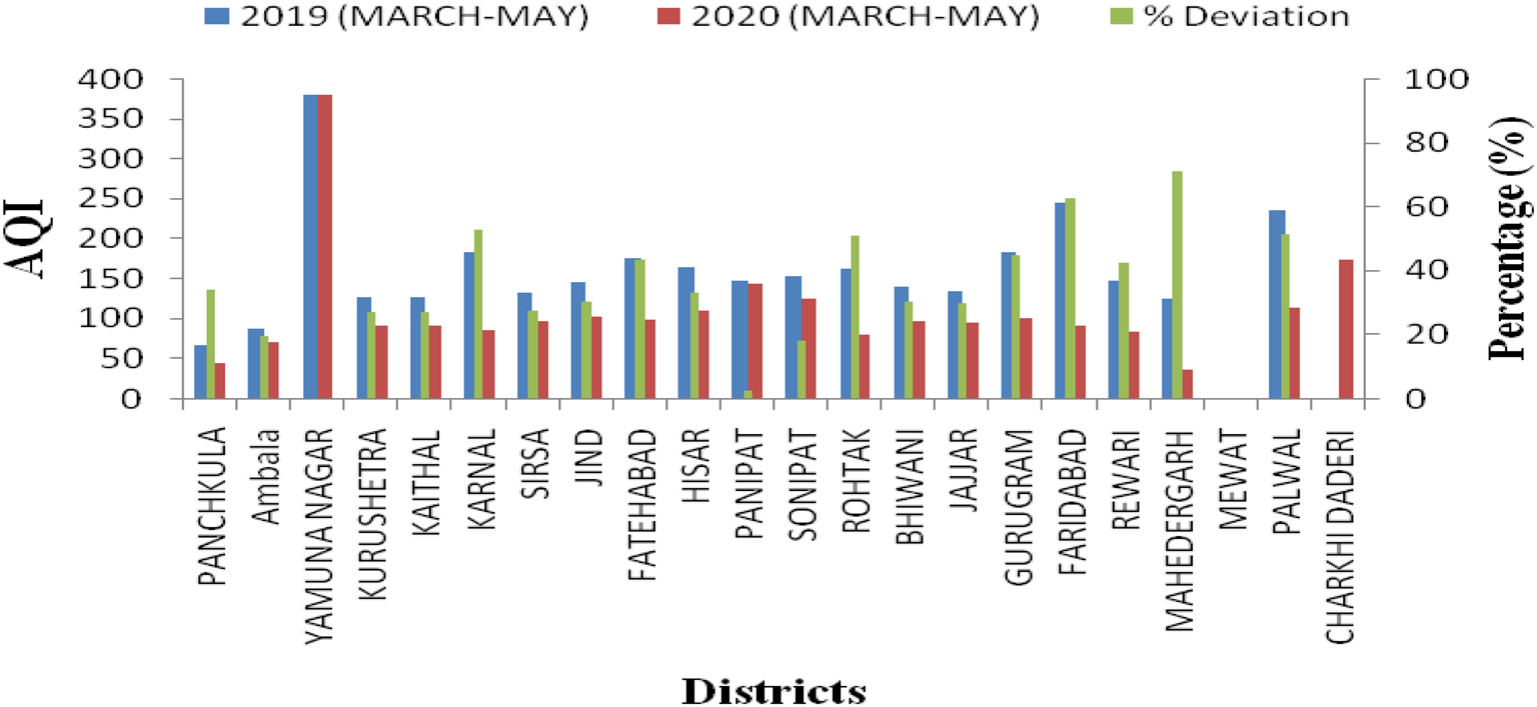
Air quality deviation observed in percentage from reference period 2019.

Figure 4 was shown that percentage deviation in air quality in 2020 as reference year 2019. Which showed that air quality was improved in 2020 during lockdown period as compared to unlock period 2019. Our results coincides with findings of Nigam et al. in Gujarat, India^24^.

## Discussion

### Spatial variation in AQI in Haryana

In 2019, the Air quality index in the eastern agroclimatic zone was showed moderated to poor quality of air in which Yamunanagar showed very poor and Faridabad and Palwal showed poor quality due to stable environmental conditions for pollutant containment float in the air and industrial, construction producing a large amount of pollutant in NCR cities and turbulence activity more during March to May period result showed by Bhankhar et al.^23^.

In 2020, the air quality index laid from satisfactory to good quality due to the lockdown period in which industry, automobile, and other construction work were not done, cyclonic activity, heat convection maximum, and unstable environment conditions from March to May. Percentage deviation was found maximum in which areas, where the air quality was so poor due to pollution sources, are automobiles, industry, constructions. Singh et al. showed similar result as COVID-19 lockdown period improved air quality in Haryana^2^. Nigam et al. revealed similar result as lockdown period improve air quality as compared to unlock period at Gujarat, India^24^.

## Conclusion

The result showed that the air quality improved during the lockdown period. The air quality was more severe in 2019 as compared to 2020 during the complete lock period. The result found that the air quality index improved in all district of Haryana in which involves most industrial area. As per study, highly developed NCR district i.e. Gurugram, Faridabad, Palwal, Karnal and Mahedergarh showed highest percentage deviation as compared to other districts. It was also find that air temperature decrease during lockdown period as per decrease the emission of green house gases. The study provides critical insights into future urban development and pollution control. Furthermore, air quality is affected by various factors, such as weather and human activities, and there is a lack of quantitative analysis on air quality. There is a need for future work to combine remote-sensed data and ground-monitored data to control extrapolation error and to explore relationships between other driving factors and air pollutants for more urban sites.

## Supplementary Information

Supplementary Figure S1.

Supplementary Information 2.

## Data Availability

Data collected from Central Control Pollution Board (CPCB) on daily basis on free of cost as available open-source at web-link https://app.cpcbccr.com/AQI_India_Iframe/.
