# Supplementary material for: Temporal and spatial impact of lockdown during COVID-19 on air quality index in Haryana, India
Source: Sci Rep. 2022 Nov 21;12:20046. doi: 10.1038/s41598-022-20885-2 (PMC9681841; doi:10.1038/s41598-022-20885-2)
Supplement: Supplementary file 1 — Supplementary Figure S1. [file 41598_2022_20885_MOESM1_ESM.docx]

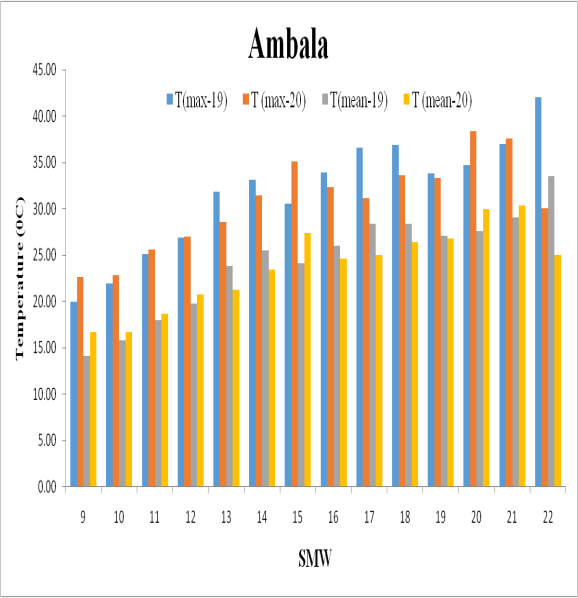

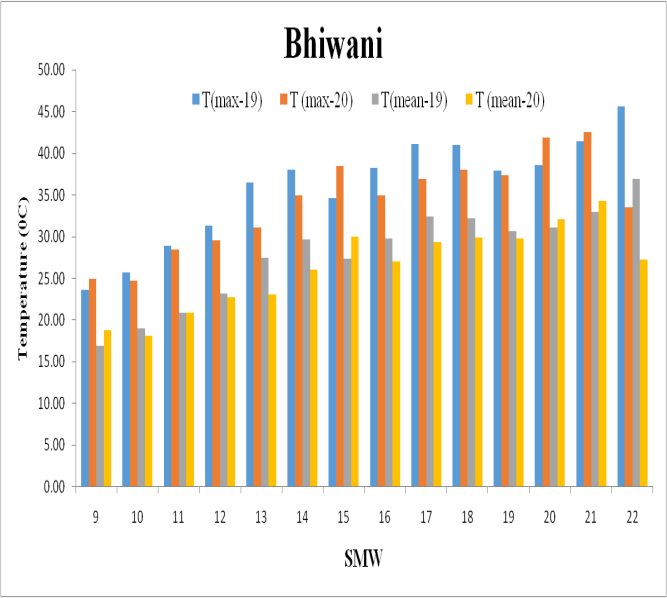

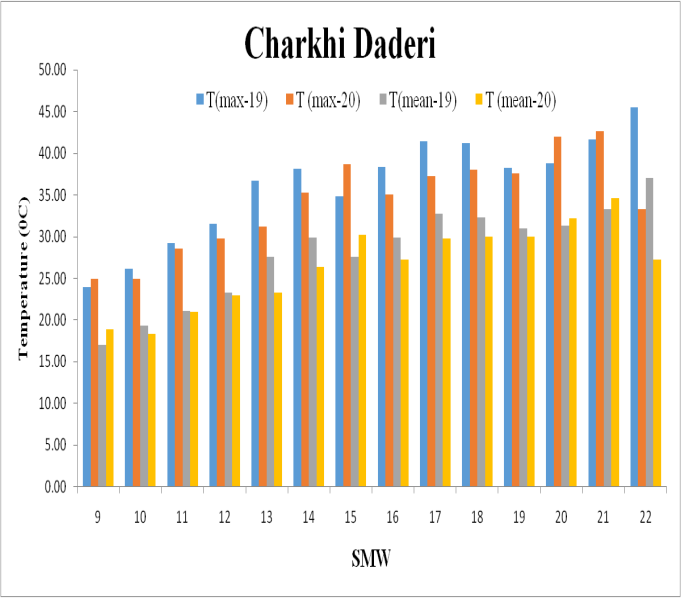

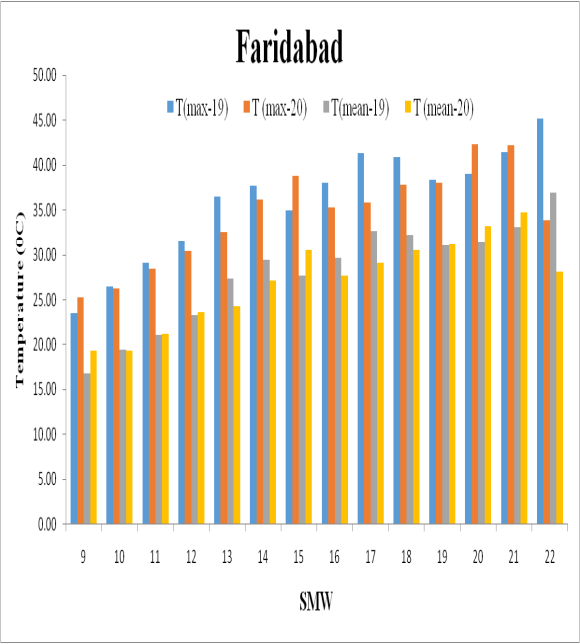

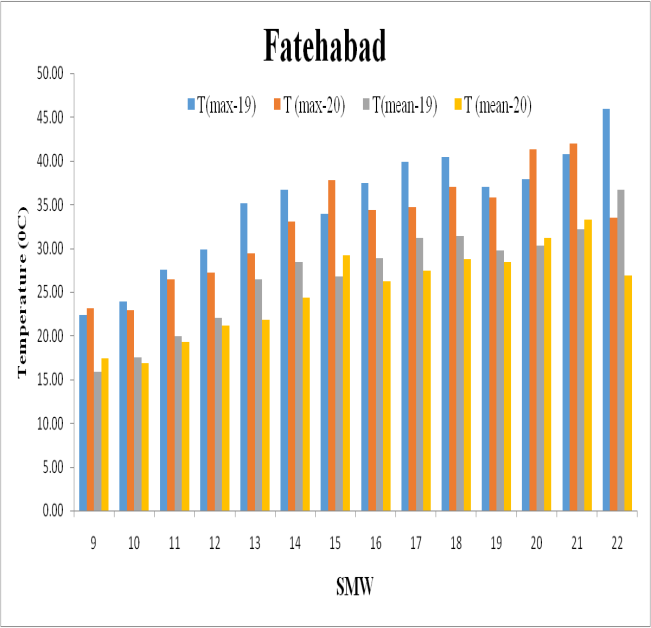

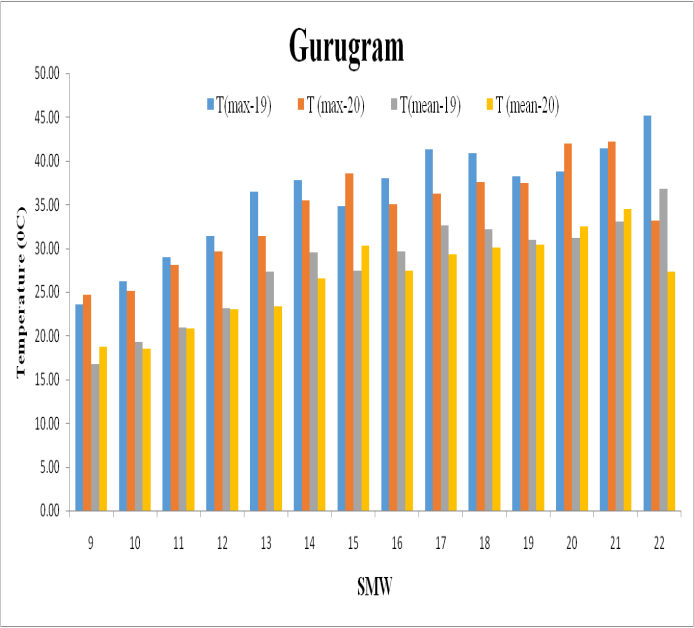

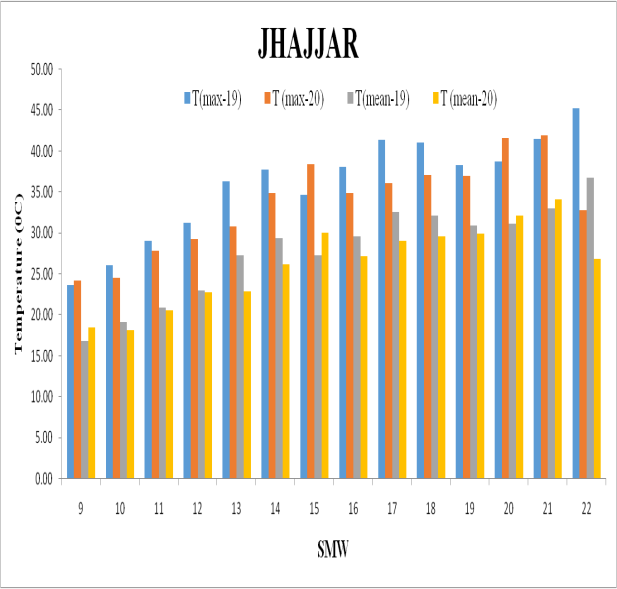

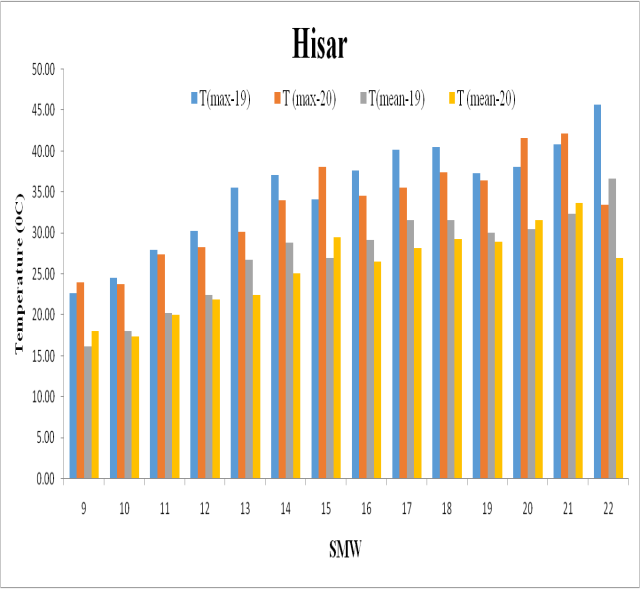

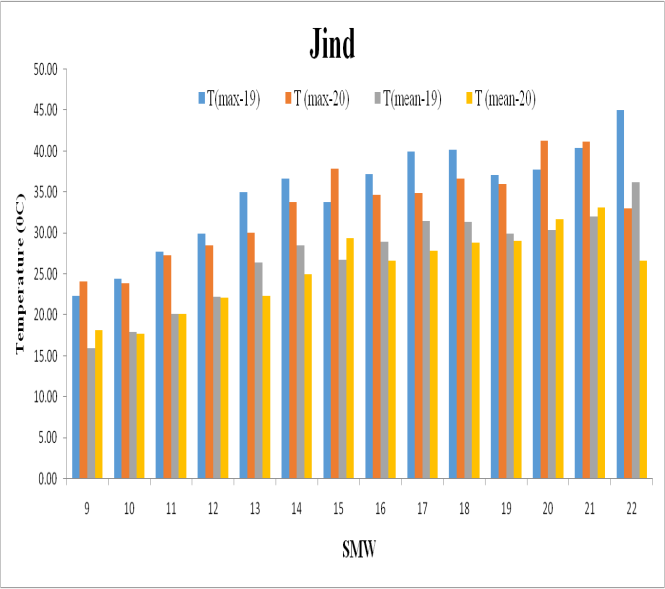

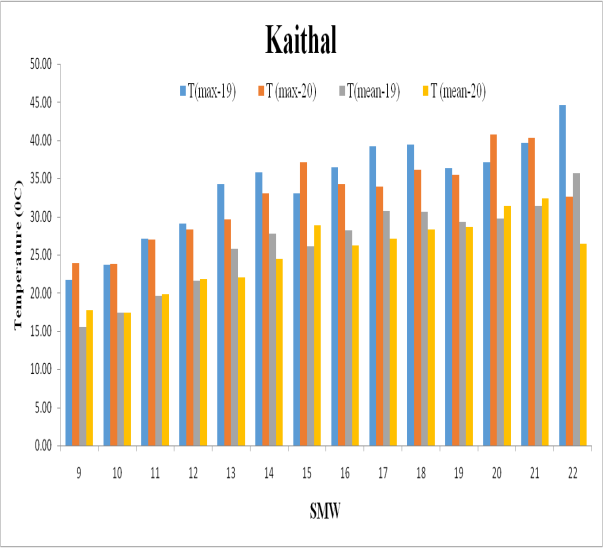

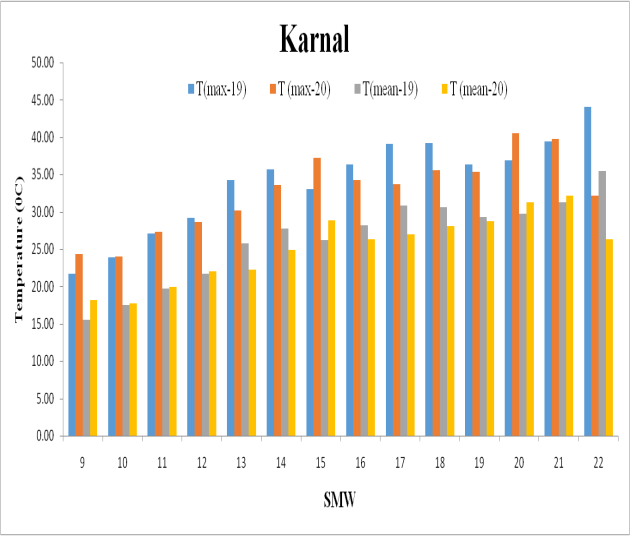

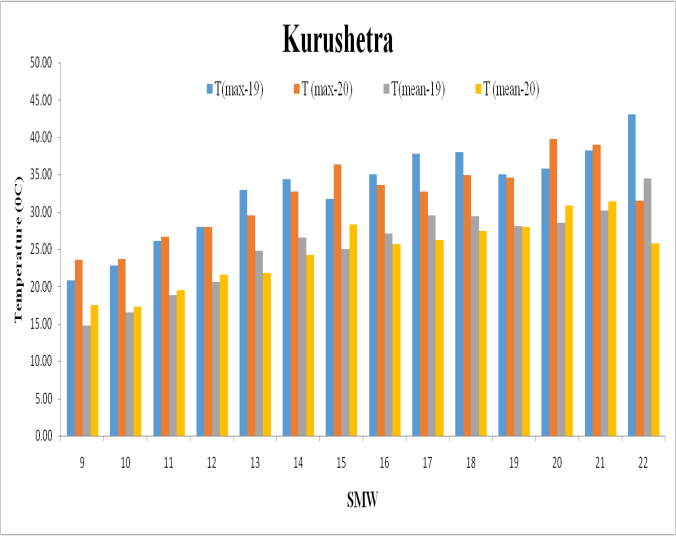

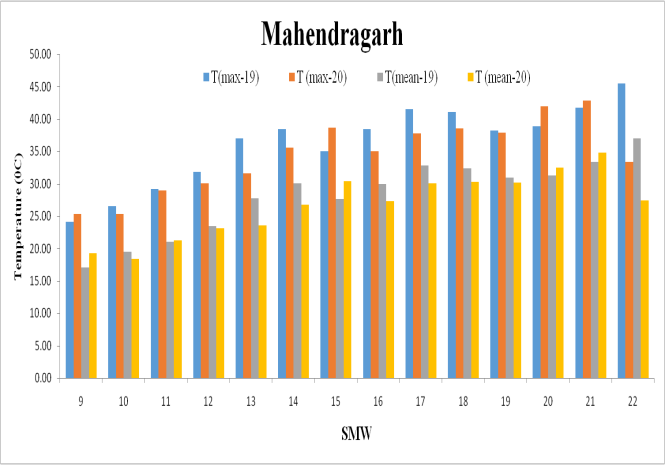

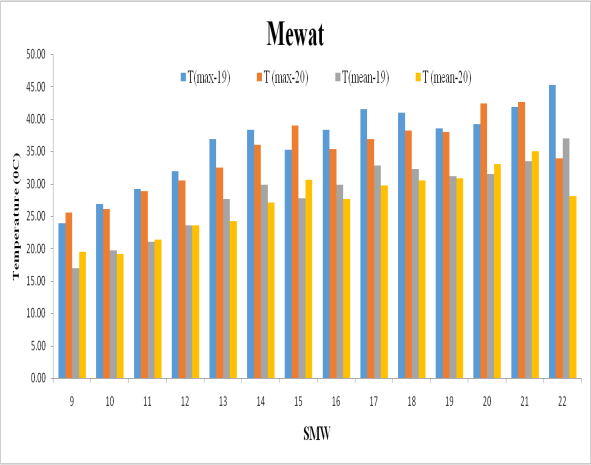

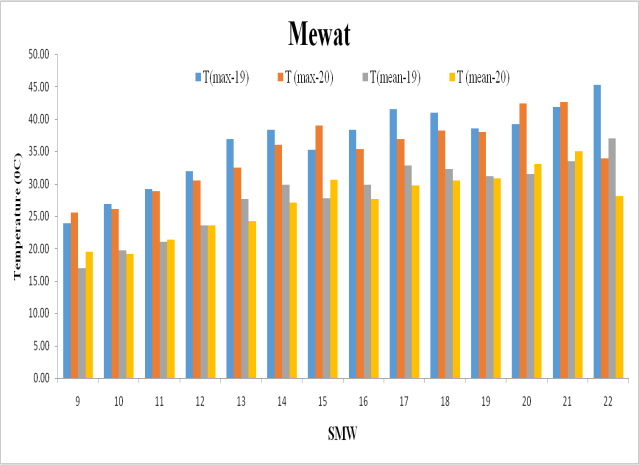

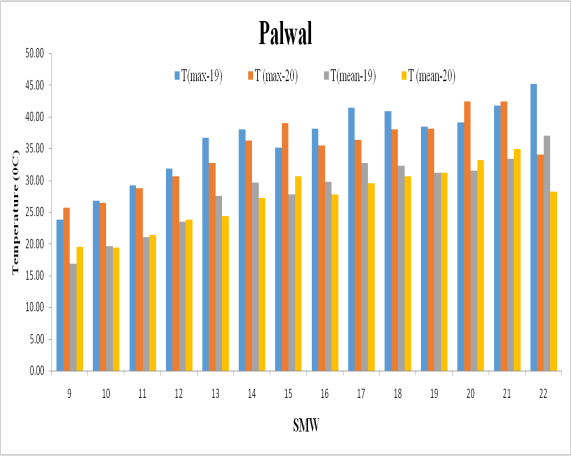

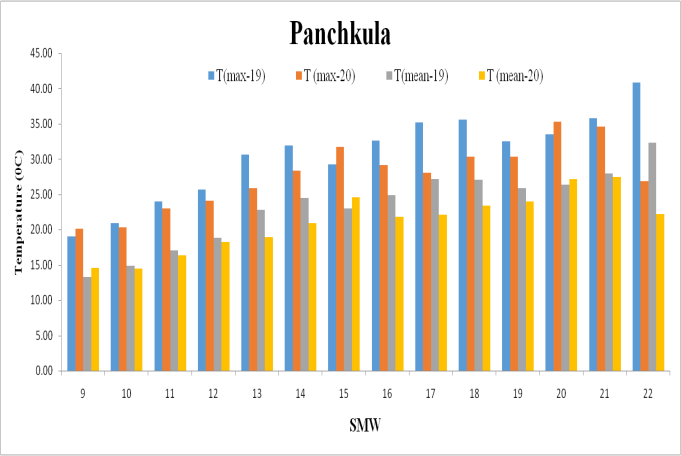

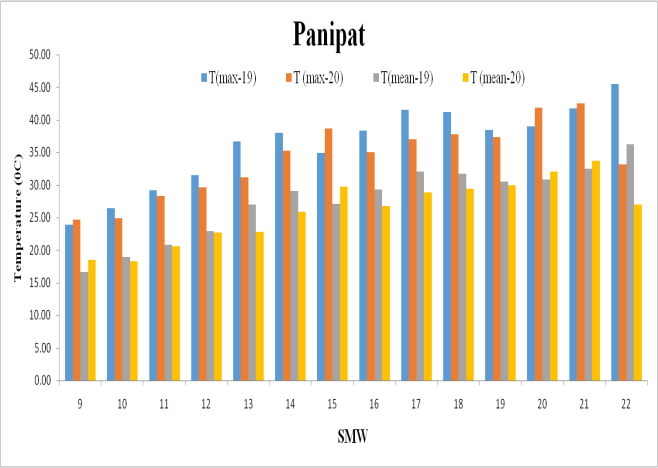

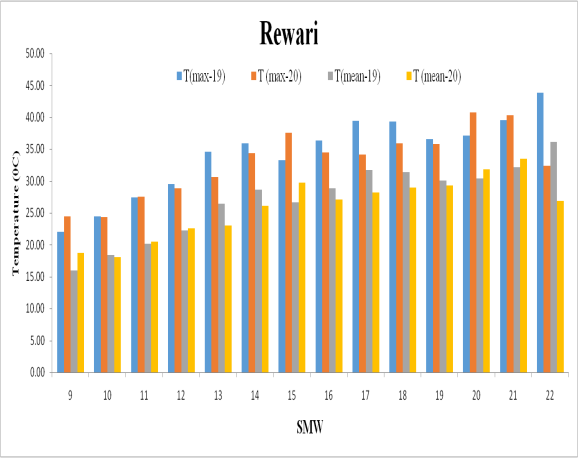

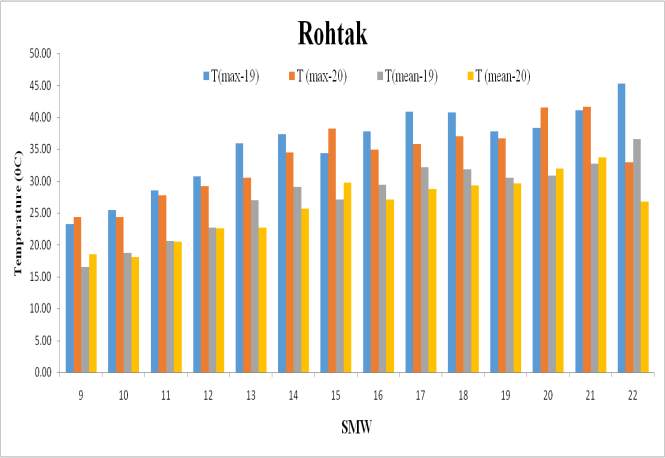

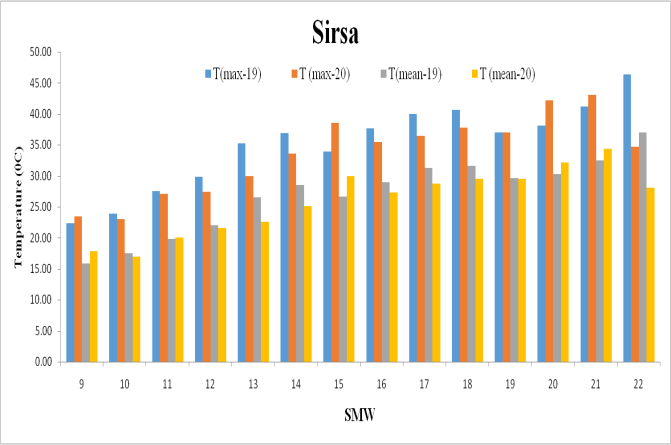

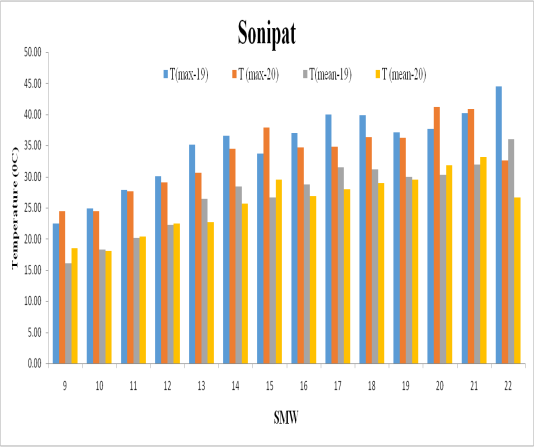

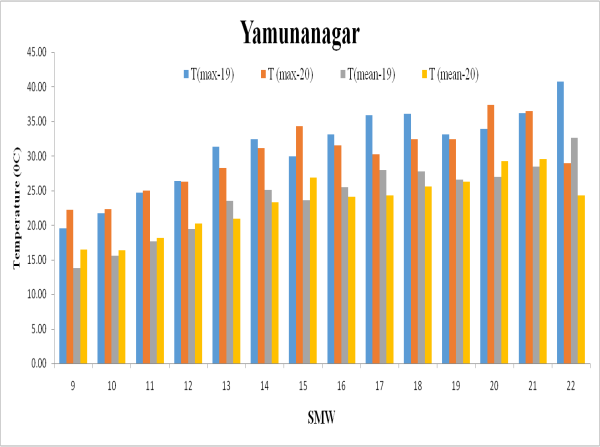

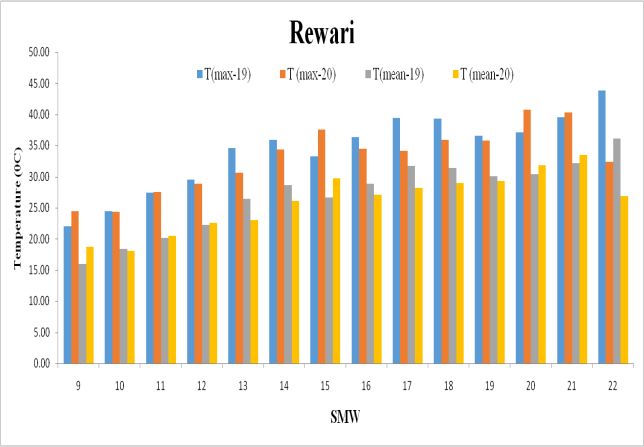

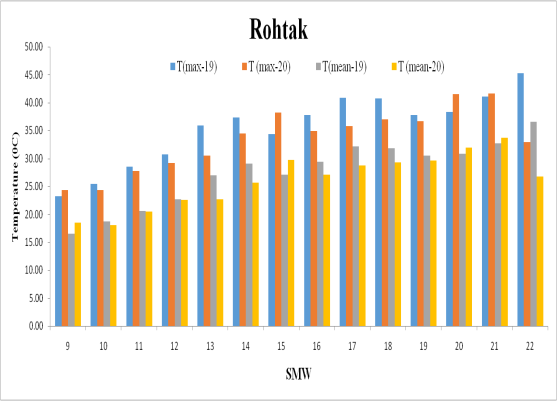

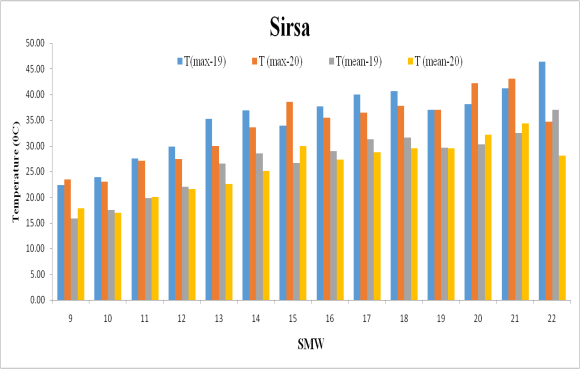

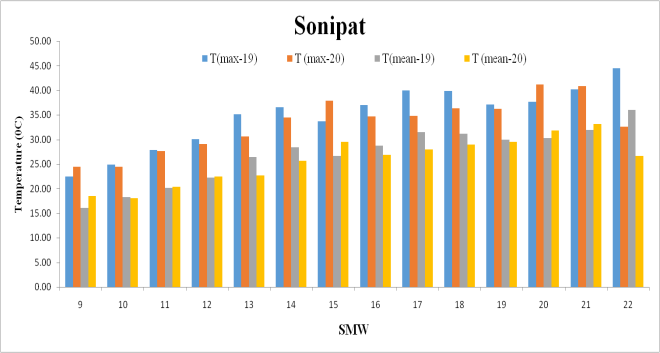

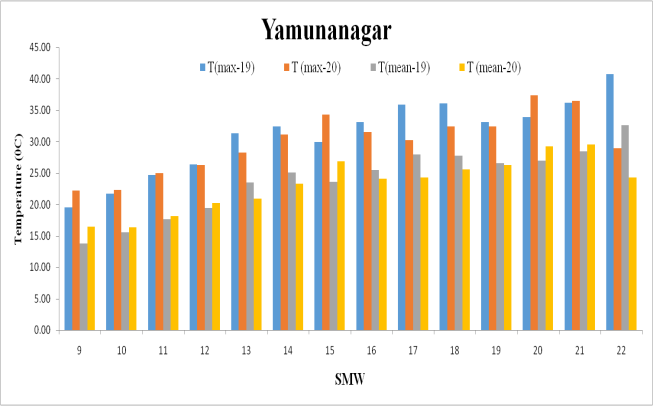


Figure S1: Graphical representation of temperature fluctuations during pre-lockdown (2019) and lock down period (2020).
