# Supplementary material for: Temporal and spatial impact of lockdown during COVID-19 on air quality index in Haryana, India
Source: Sci Rep. 2022 Nov 21;12:20046. doi: 10.1038/s41598-022-20885-2 (PMC9681841; doi:10.1038/s41598-022-20885-2)
Supplement: Supplementary file 2 — Supplementary Information 2. [file 41598_2022_20885_MOESM2_ESM.docx]

| Data set derived from CPCB for AQI in Haryana state, India | | | |
| --- | --- | --- | --- |
| Districts | 2019 (AQI) | 2020 (AQI) | % Deviation |
| PANCHKULA | 66 | 44 | 33.89651 |
| AMBALA | 88 | 71 | 19.3328 |
| YAMUNA NAGAR | 380 | 380 | 0 |
| KURUSHETRA | 126 | 92 | 27.15276 |
| KAITHAL | 126 | 92 | 27.18566 |
| KARNAL | 184 | 86 | 53.0152 |
| SIRSA | 133 | 97 | 27.2715 |
| JIND | 146 | 102 | 30.11837 |
| FATEHABAD | 175 | 99 | 43.57586 |
| HISAR | 165 | 110 | 33.19165 |
| PANIPAT | 147 | 143 | 2.691831 |
| SONIPAT | 152 | 125 | 18.11006 |
| ROHTAK | 163 | 80 | 50.86021 |
| BHIWANI | 140 | 97 | 30.40341 |
| JAJJAR | 135 | 95 | 29.64457 |
| GURUGRAM | 183 | 101 | 44.91275 |
| FARIDABAD | 246 | 91 | 62.85106 |
| REWARI | 147 | 84 | 42.69859 |
| MAHEDERGARH | 125 | 36 | 71.17099 |
| MEWAT | 0 | 0 | 0 |
| PALWAL | 235 | 115 | 51.30295 |
| CHARKHI DADERI | 0 | 173 | 0 |

**Supplementary Information**
